# Impact of Ag Nanoparticles (AgNPs) and Multimicrobial Preparation (EM) on the Carcass, Mineral, and Fatty Acid Composition of *Cornu aspersum aspersum* Snails

**DOI:** 10.3390/ani11071926

**Published:** 2021-06-28

**Authors:** Tomasz Niemiec, Andrzej Łozicki, Robert Pietrasik, Sylwester Pawęta, Anna Rygało-Galewska, Magdalena Matusiewicz, Klara Zglińska

**Affiliations:** 1 Division of Animal Nutrition, Institute of Animal Sciences, Warsaw University of Life Sciences, Ciszewskiego 8, 02-786 Warsaw, Poland; andrzej_lozicki@sggw.edu.pl (A.Ł.); anna_rygalo-galewska@sggw.edu.pl (A.R.-G.); klara_zglinska@sggw.edu.pl (K.Z.); 2 Institute of Materials Science and Engineering, Lodz University of Technology, Stefanowskiego 1/15, 90-924 Lodz, Poland; robert.pietrasik@p.lodz.pl (R.P.); sylwester.paweta@p.lodz.pl (S.P.); 3 Hart-Tech Sp. z o.o., Niciarniana 45, 92-320 Lodz, Poland; 4 Department of Nanobiotechnology, Institute of Biology, Warsaw University of Life Sciences, Ciszewskiego 8, 02-786 Warsaw, Poland; magdalena_matusiewicz@sggw.edu.pl

**Keywords:** silver nanoparticles, multimicrobial preparation, biosecurity, nanotoxicity, *Cornu aspersum aspersum*, snails

## Abstract

**Simple Summary:**

Microbial control agents kill or inhibit the growth of pathogenic microbes, but they should also be safe to use for farmed animals. The techniques for safely eliminating microorganisms also apply to the quality of the animal product. The study analysed the effects on basic chemical composition, mineral content, and fatty acid profiles in the bodies of *Cornu aspersum aspersum* snails, of the application of paint containing silver nanoparticles (AgNPs) and/or a microbiological preparation (EM) on feed tables. The use of nano-Ag paint increased Ag, Zn, Fe, and Ca retention, and remodelled the fatty acid profile. EM did not affect the fatty acid profile but increased the retention of Fe, Cu, P, Mg, and Zn in the carcasses. It was concluded that EM is a safer and more beneficial agent for controlling microorganisms. The obtained results indicated the toxicity of nano-Ag paint.

**Abstract:**

The hygienic practices on farms should reduce pathogenic microorganisms while simultaneously not harming the animals themselves; they must also not degrade the products’ quality. We assessed the effect of covering feed tables with paint containing silver nanoparticles (AgNPs) and the periodic spraying of effective microorganisms (EM) on production indicators and basic chemical composition, mineral content and fatty acid profiles in the bodies of *Cornu aspersum aspersum* snails. The animals were divided into four groups: (1) control, (2) with feed tables covered with AgNPs paint, (3) with EM spray applied and (4) with both factors—AgNP paint and EM spray. The highest increase in Ag, Zn, Fe and Ca retention, and the remodelling of the fatty acid profile in the carcasses of snails was found to be in the group of animals in contact with the feed tables covered with AgNP paint. In the group of animals exposed to the action of EM, an increased retention of Fe, Cu, P, Mg and Zn was found.

## 1. Introduction

According to estimated data, an increase in snail consumption is expected to reach 50 thousand tons by 2025. The highest consumption of the meat of these molluscs is recorded in Spain, Morocco and France. Morocco played a crucial role in the produce market in 2019 (17.4% of world exports), while France and Spain are the largest snail importers (23.4% and 20.4% of global imports, respectively) [1].

One of the aspects of industrial snail farming is maintaining the microbiological regime of the breeding park environment. The long-term use of breeding parks and the rotting remains of concentrated feed mixtures lying on feed tables favour the development of pathogenic microflora [2]. As reported in the literature, the snails’ intestinal microflora include several opportunistic pathogens, such as *Enterobacter*, *Pseudomonas* and *Aeromonas* [3]. Diseases caused by *Pseudomonas* spp., especially *Pseudomonas aeruginosa*, cause intestinal infections in snails and disrupt the normal growth and development processes of these animals [4]. In 1994, snail plantations in France suffered severe economic losses due to massive deaths of *Cornu aspersum aspersum* farm snails. First, Kodjo et al. [5] and then Kiebre-Toe [6] demonstrated that the bacterium *Aeromonas hydrophila* was the cause of this epizootic. Dushku et al. [7] isolated the pathogen *Listeria monocytogenes* from the gastrointestinal tract of snails, which, with high hypervirulence, can cause dysbiosis in intensively bred snails.

Biosecurity is defined in the OIE Terrestrial Animal Health Code as a set of management and physical measures designed to reduce the risk of introduction, establishment and the spread of animal diseases, infections, or infestations to, from, or within an animal population [8]. The annual fertilisation of soil with calcium carbonate is practised on snail farms to reduce the occurrence of harmful bacteria and moulds living in acidic soils [9]. It is a cheap and effective process for maintaining proper microbial homeostasis in the environment, although it may be insufficient in intensive breeding situations. Sterilisation of the topsoil, regular removal of food debris from feed tables and fumigation of the farm environment to eliminate microorganisms, parasites and predators, are recommended [2,10]. Effective microorganisms (EM) probiotic preparations are often used in breeding practice and is a mixture of beneficial microorganisms that occur naturally in nature [11]. The EM concept was developed in 1980 by T. Higa of the University of Ryukyus (Okinawa, Japan). EM’s primary goal is to restore healthy ecosystems to both soil and water using mixed cultures of beneficial and naturally occurring microorganisms. In animal studies, the effect of EM on improving production and health-promoting effects was demonstrated by modulating the composition of gastrointestinal microflora, stimulating the proliferation of the intestinal epithelium and regulating the animal’s immune response [12,13,14]. In addition, EM improves soil quality and fertility, as well as crop growth and quality by improving soil structure, increasing microbial balance and increasing both nutrient availability and plant nutrient uptake [15,16]. Moreover, EM’s applicability as a biological technique to mitigate the adverse effects of excess salt in soil has been demonstrated [17].

In recent years, many hygiene practices in animal production have begun to be based on silver nanoparticles. Currently, thanks to their antimicrobial properties [18,19], silver nano-particles are used in animal production as disinfectants and for the reduction of ammonia and nitrogen oxide emissions [20]. Silver nano-particles introduced into air filtration systems [21], applied directly to surfaces [22], or as a component in paint [23], stabilize the microbiological flora of closed spaces. Research by Dobrzański et al. [24] demonstrated that the use of nano-silver as a supplemental biocidal preparation for plant litter in broiler houses produced positive antimicrobial results each time the plant litter was changed. Depending on the concentration, size, shape and method of administration, silver nano-particles have been responsible for modulating the immune system and inflammation [24] and influencing quantitative and qualitative changes in the intestinal microbiome in animal studies [25,26]. Despite its high antimicrobial potential, silver nano-particles may also be a source of silver ions with greater toxicity [22,27] and may accumulate in tissues [28] and induce oxidative stress and inflammation [29].

Techniques to prevent infections in snails should limit the impact of undesirable biotic factors, but, at the same time, should avoid accumulating toxic substances or adversely affecting the quality features of the products obtained from them. Easily digestible snails’ meat can be an alternative to other farm animal products: it has a low-fat content [30,31], which translates into a low energy value; 60–80 kcal/100 g [32]. Its dietary properties are also determined by the high biological value of its protein, which is a good source of exogenous amino acids [31,33,34]. Snails constitute 35.5% of all amino acids and the levels of histidine, leucine and threonine are higher than in beef, fish or chicken [35]. The proportion of protein in the dry matter of snail meat is 73% [30]. Another advantage of these animals’ meat is the large share of unsaturated fatty acids in the total fat fraction—from 45% to 80% [31,36,37]. The meat of these molluscs is also a source of minerals (100 g of fresh snail meat covers about 60% of the daily requirement for calcium and 25% of the daily requirement for iron in an adult human) and B, A and K vitamins [30,36,38].

Our goal was, therefore, to assess the impact of the environmental additives used in biosecurity on the chemical composition, fatty acid profile and mineral content in carcasses of *Cornu aspersum aspersum* snails kept in intensive breeding situations.

## 2. Materials and Methods

### 2.1. Animals and Experimental Design

**Figure 1:**
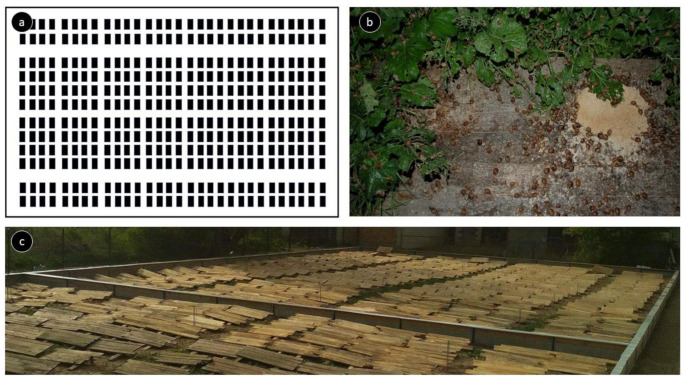
Feed tables: (**a**) visualisation of one plot, (**b**) snails with feed (mixture on the table and *Brassica rapa* growing around), (**c**) plots with tables in the experimental plot.

The research was carried out during the farming period, in the months of June to September 2018, on a *Cornu aspersum aspersum* snail farm. One control group and three treatment groups were used with two replicates plots for each group. The individual plot areas were 625 m^2^, with an average number of 350 snails per m^2^. There were 360 feed tables, made of beech boards, in each plot, each with an area of 0.6 m^2^ (Figure 1). The tables’ surfaces were covered with a paint intended for surfaces in contact with food—a solvent-free epoxy system.

In the control and EM groups, the prepared feed tables were covered with paint without the addition of nano-Ag. In two subsequent groups (AgNPs and AgNPs + EM groups), the feed tables were covered with the same paint as in the control group, but with added nano-Ag in the amount of 100 mg/L (AgNPs). Additionally, in the EM and AgNPs+EM groups, the feed tables were covered with the preparation of EM Bokashi^®^ at a concentration of 10%.

The paint containing the nano-Ag was obtained by adding the nano-silver formulation “Al 2100” (50–60 nm silver nano-particles, polyvinyl alcohol (PVA) coating, alcohol suspensions, concentration 1000 ppm, viscosity 1 ± 0.2 mPas, thixotropy index ~1.0, specific gravity 0.8 ± 0.2 g/cm^3^ from Amepox, Łódź, Poland) to the solvent-free epoxy. The EM Bokashi^®^ preparation used in the experiment was manufactured by a commercial company (Greenland Technologia EM, Janowiec, Poland). It contains a mixture of microorganisms, as described by Laskowska et al. [39], namely, *Saccharomyces cerevisiae* (Y200007) 5 × 10^4^ CFU/g, *Lactobacillus casei* (ATCC 7469) 5 × 10^8^ CFU/g, *Lactobacillus plantarum* (ATCC 8014) 5 × 10^8^ CFU/g, *Enterococcus faecalis* (UC-100 (CGMCC No.1.0130)) 2.5 × 10^6^ CFU/g, *Enterococcus faecium* (NCIMB SF68) 5 × 10^9^ CFU/g, *Bifidobacterium bifidum* (ATCC 29521) 5 × 10^8^ CFU/g, *Bifidobacterium pseudolongum* (ATCC 25526) 5 × 10^8^ CFU/g, *Bacillus licheniformis* (DSM 5749) 4 × 10^9^ CFU/g, *Bacillus cereus var. toyoi* (NCIMB 40112) 4 × 10^9^ CFU/g, *Bacillus subtilis* (MA139) 4 × 10^11^ CFU/g and *Clostridium butyricum* (MIYAIRI 588 (CBM588)) 1 × 10^8^ CFU/g.

The EM Bokashi^®^ preparation, in the form of an aqueous suspension, was spread on the feed tables’ surface and under the tables using a hand-held sprayer. During the entire study period (June to September 2018), the preparation was used once a week on Mondays at the fixed time of 8:00–10:00.

The snails had access to *Brassica rapa* var. *sylvestris* fodder in all the plots. They were fed a concentrated mixture that included corn, wheat, extracted soybean meal, corn dried distillers’ grains with soluble (DDGS), fodder yeast, oil, fodder chalk, 1-calcium phosphate, NaCl and premix. The nutritional value of 1 kg of the mixture was as follows: crude protein, 170 g; lysine, 9 g; methionine + cysteine, 4.8 g; crude fibre, 33 g; crude fat, 50 g; Ca, 130 g and P, 7.5 g. The animals had constant access to concentrated feed, which was given twice a day at 9:00–10:00 and again at 17:00.

Green fodder samples were collected once a month and their basic composition was analysed. On average, over the entire period of the experiment, the basic composition of green fodder was as follows: dry matter, 164 g; crude protein, 191 g/kg dry matter (DM); crude fibre, 186 g/kg DM; neutral detergent fibre (NDF), 394 g/kg DM; crude fat, 42 g/kg DM; crude ash, 134 g/kg DM; Ca, 26 g/kg DM and P, 7.2 g/kg DM. The remaining leftovers and snail droppings were removed from the tables before the fresh feed was distributed. As a standard procedure used on the farm for all the plots, an automatic field sprinkler system was installed, ensuring a relative humidity of 85–95% between 21:00 and 3:00. 

### 2.2. Experimental and Analytical Procedures

Two-day-old snails were introduced to the experimental plots at the beginning of June. A total of 160 snails, 20 per one plot, were taken at the end of the feeding trial for carcass evaluation. The snails were starved overnight and euthanized by fast-freezing them at −20 °C for 30 min. The carcasses were removed from the shell with tweezers and weighed whole. Finally, the carcass samples were frozen and stored at −80 °C for further analysis.

The chemical composition of the snail carcasses was determined according to AOAC (Association of Official Analytical Chemists) guidelines. [40]: dry matter by drying at 105 °C to constant weight, crude ash by incineration at 550 °C for 6 h, crude protein (N × 6.25) by using the Kjeldahl technique (Kjeltec System 1026 Distilling Unit, Foss Tecator, Sweden) and crude fat after extraction with petroleum ether using the Soxhlet method.

The P, Ca, Mg, Cu, Zn, Fe and Ag content was determined after mineralising a sample in a chamber furnace at 450 °C for 12 h and dissolving the residue in diluted (1:2) nitric acid. The content of the mentioned elements was determined in the solutions by using inductively coupled plasma atomic emission spectrometry (ICP-OES, Perkin Elmer ELAN DRC II). 

Thiobarbituric acid reactive substances (TBARS) were expressed as equivalents of malondialdehyde (MDA) and MDA precursor (1.2.3.3-tetraethoxypropane (TEP)) was used as a standard to plot a standard curve. Absorbance was read at a wavelength of 532 nm using a Tecan Infinite M200 spectrophotometer. TBARS were determined according to the methodology presented by Uchiyama and Mihara [41].

Total lipids from the snails’ tissues were extracted following the procedure described by Folch et al. [42]. The fatty acid composition was determined by gas chromatography by comparing their retention times with the corresponding standard (Sigma-Aldrich, SA, Alcobendas, Madrid, Spain). The lipid extracts were esterified with a mixture of boron trifluoride, hexane and methanol [43]. Fatty acid methyl esters were analysed using a Chromapack CP 9001 Gas Chromatograph (Chrompack Instrumental BV) equipped with a WCOT fused silica capillary column and a flame ionisation detector. The monounsaturated (MUFA), polyunsaturated (PUFA) and saturated (SFA) fatty acids’ content were also calculated.

**Figure 2:**
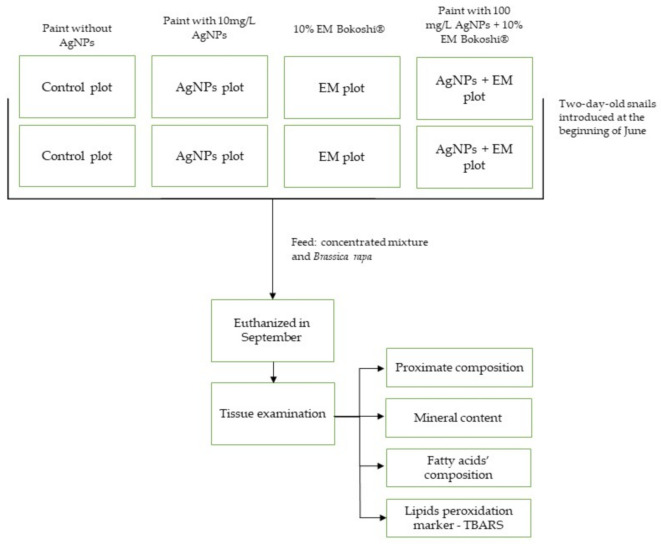
Experiment design.

The experiment’s design is presented in Figure 2.

### 2.3. Statistical Analysis

The results were analysed using one-way analysis of variance (ANOVA) and Duncan’s multiple range test, using StatGraphics 4.1 Plus (StatPoint, Inc., Warrenton, VA, USA). A difference of *p* < 0.05 between means was considered to be significant.

## 3. Results

The analysis of the snail carcasses’ basic chemical composition indicates an increased proportion of ether extract due to the simultaneous use of both biosecurity methods (AgNPs + EM). The microbiological preparation and the nano-Ag paint, when applied separately, did not change the snails’ basic chemical composition compared to the control group (Table 1).

Our results indicate that the paint containing AgNPs and the EM influenced the minerals content of the snails’ bodies. The animals exposed to the AgNP paint had increased Ca, Fe, Zn and Ag content in their carcasses compared to the control group. Among all the groups, this group had the highest content of Ca and Zn. In turn, this group was characterised by the highest retention of Ag. Except for Ca and Ag the mineral content increased in the EM group compared to the control group EM resulted in the highest retention of Fe, Mg and P among all the experimental groups. However, the lowest retention of Ag was found in the same group. Increased retention of Cu, Fe, Mg and Zn was observed in the animals exposed to AgNPs + EM. The Ca and P content of the snails’ carcasses in this group was the lowest among all the groups (Table 2).

We found that the paint containing AgNPs increased the proportion of C16:1 and C18:2 n-6 fatty acids in the fat of snails. The same result was observed in the AgNPs + EM group, where 16- and 18-carbon acids (C16:0; C18:1) were significantly increased compared to the control group. Additionally, the content of the sum of MUFA and eicosatrienoic acid in fat was the highest in this group, among all the experiment groups. Simultaneously, in the AgNPs and AgNPs + EM groups, a reduced proportion of 20- and 22-carbonic acids (C22: 0, C20: 1, C20: 2, C20: 3 n-6) was found in the fat compared to the control group. In the groups associated with the AgNP paint, an increased proportion of compounds that reacted with thiobarbituric acid in the snail carcasses was found. There was no observable effect of EM on changes to the fatty acid content in the snail carcasses (Table 3).

## 4. Discussion

There were two methods of biosecurity used in this work. The first used paint containing Ag nano-particles, whose task was to stabilise the microflora on the feed tables. The second method used was spraying a microbiological preparation with a broad spectrum of activity against the microflora in the external environment and the digestive tract environment of the tested animals. As noted [44], the methods used achieved the desired effects in maintaining microbiological homeostasis on the feed tables. However, the interaction of the paint containing silver nano-particles and the microbiological preparation spray showed high variability. It strongly differentiated the research groups in terms of the snails’ chemical composition.

### 4.1. Chemical Composition

The chemical composition of the snail carcasses changed only in the AgNPs + EM group. The increased proportion of fat in animals exposed to the two experimental factors can only be explained by the paint and EM interaction’s mutual synergy. In studies on porkers, the addition of lactic acid bacteria to the feed did not affect the chemical composition of the carcasses [12]. However, in other studies, the same author observed a lower fat content in pigs that received the same probiotic [45]. Few studies consider the effect of silver on the chemical composition of animal products. Pineda et al. [46] reported that broiler treated with silver nanoparticles supplied in drinking water retained more nitrogen (N) per kg metabolic body size. The results demonstrated that AgNPs affect N utilisation; however, it does not influence the growth performance of chickens. Saleh and El-Magd [47] found no changes in the weight of abdominal fat in broilers fed with silver, both in nano-particle and ionic forms. In studies on carp fry, it was found that the addition of silver nanoparticles to water increased the fat and protein content in the carcasses of these animals [48]. Yue et al. [49] found that injected silver nano-particles promoted obesity by inhibiting beige adipocytes’ differentiation and function in mice.

### 4.2. Mineral Composition

The AgNP paint and the EM both affected the retention of the feed’s minerals in the snail carcasses. The study has demonstrated that the silver in the paint can be transmitted to animal tissues. The diffusion of silver ions released from the surface of the nanoparticles and their accumulation in the organisms has been confirmed by many authors [50,51]. Additionally, it is worth emphasising the increased retention of Zn in groups of animals exposed to silver. This may be related to the influence of silver compounds on the expression of metallothioneins (MT) and their zinc accumulation. Many studied mollusc species are effective indicator organisms, accumulating high concentrations of metals and metalloids, depending on the concentration gradient of these compounds in the surrounding environment [52]. Heavy metals (Cu, Zn, Cd, Ag, Hg) can induce MT synthesis in vertebrates and invertebrates [53]. MT plays a role in Cu and Zn’s basic metabolism and the detoxification of excessive intracellular amounts of these metals and the detoxification of unnecessary and dangerous Cd, Ag and Hg [52]. In studies on rats, MT concentrations were increased in the liver and kidneys under the influence of Ag^+^ ions [54]. It is believed that an increase in MT expression may buffer silver ions, thus limiting Ag^+^ mediated cell damage [55].

Furthermore, Ag^+^ binds to MT with high affinity and displaces Zn^2+^ [56]. It can be assumed that the silver that was contained in the paint and absorbed into the snails’ gastrointestinal system in the form of Ag^+^ ions, increased the expression of MT. On the one hand, the increased MT pool protected the animals against the silver, but, on the other, influenced the increased absorption and accumulation of zinc in protein transporters. However, it cannot be ruled out that the zinc displaced by Ag^+^ from MT was present in the free state. Cortese-Krott et al. [55] reported that AgNO_3_ induces a transient increase in intracellular free Zn^2+^ in human fibroblasts. In the present experiment, the Fe content was also increased in animal carcasses in contact with the nano-Ag paint. As Minghetti and Schirmer [27] point out, silver strongly destabilises Zn and Fe homeostasis, leading to reduced content inside fish cells in in vitro tests. Thus, one cannot rule out that an extracellular accumulation of these elements occurs under Ag’s influence, which increases the Zn and Fe content in the whole carcass. On the other hand, Ochoa-Meza et al. [57] demonstrated an increased innate immunity in *Penaeus vannamei* shrimps under silver nano-particle treatment. The results they obtained suggest a mechanism whereby AgNPs might compete with the white spot syndrome virus’ attachment to the cell, occupying the interaction sites of the pathogen-associated, molecular pattern (PAMPs) recognition proteins and triggering an immune response to cope with virus proliferation.

The experiment showed the effect of EM on the retention of many elements, including Cu, Fe, Mg and P, which can be explained by the higher availability of these elements inside the snails’ gastrointestinal tract. Minerals in green forage are surrounded by phytate [58]. Phytate reduces the availability of such minerals as Zn, Fe, Cu, Ca and P. Bacteria and fungi produce the phytase enzyme, which can release minerals degraded by phytates [59,60]. Moreover, some probiotic bacteria produce peptides with biological functions that can increase mineral availability [61]. There are reports on phytase-producing probiotics belonging to the *Lactobacillus*, *Enterococcus* and *Bifidobacterium* genera [62]. Askelson [63] investigated the administration of recombinant *Lactobacillus* spp. *L. acidophilus, L. gasseri* and *L. gallinarum* carrying *Bacillus subtilis* phytase in broilers, which improved phytate utilisation. Al-Bishri et al. [64] studied the effects of a diet containing phytase from *Lactobacillus acidophilus* on bone performance in albino rats. A pronounced increase in the level of phosphorous, calcium and zinc in bone and serum was observed. The enzyme treated diet was even better than the zinc supplemented diet. While the gastrointestinal tract of snails contains phytase-producing fungi [65] that enable phytate degradation, in this study, additional hydrolysis of phytates by the EM enzymes could have increased the pool of available minerals in the intestinal lumen. In the AgNP + EM group, silver seems to suppress the effect of the increased bioavailability of elements, which can be explained by the reduction of gastrointestinal microflora by silver.

### 4.3. Profile of Fatty Acids

Many studies have analysed the effect of trace elements, especially heavy metals, on the fatty acid profile of algae and some marine invertebrates [66,67,68]. It is known that changes in the activity of fatty acid biosynthesis mechanisms are typical biochemical reactions to pollutants and their accumulation in marine organisms [67]. In this study, the fatty acid profile changed mainly in the carcasses of snails that were in contact with feeding tables covered with silver nano-particle paint. In each of the analysed groups, there was a tendency for the share of some 16-carbon SFA (palmitic acid), MUFA (palmitoleic acid) and PUFA (linoleic acid) to increase, while, at the same time, there was a reduction in the amount of 22-carbon SFA (behenic acid), MUFA (eicosenoic acid) and PUFA (eicosadienoic acid, dihomo-γ-linolenic acid) compared to the control group. It is suspected that silver, in displacing zinc with MT, reduced this element’s availability for many biochemical reactions. As is known, Zn acts as a cofactor for the FA desaturase and elongase enzymes [69,70] which convert linoleic acid (LA) and α-linolenic acid (ALA) into their long-chain metabolites–PUFA. Studies by other authors suggest that Zn deficiency increases the share of saturated and monounsaturated fatty acids and significantly reduces the polyunsaturated fatty acid profile in hepatic diglycerides [71]. Chimhashu demonstrated that Zn deficiency might impede the rate of the desaturase reaction necessary for converting LA to dihomo-γ-linolenic acid (DGLA) [72]. PUFA synthesis can also occur in less complex animals de novo [73]. The presence of fatty acid desaturases and elongases have been observed in molluscs [74]. Our study found an exceptionally high LA and low DGLA content in groups of snails that were in contact with paint enriched with Ag nano-particles. Xiang et al. [75] investigated the toxic effects of a series of sublethal concentrations of AgNPs (0.25–1.25 mg/L) on the membranes of the gills of freshwater carp (*Cyprinus carpio*), based on changes in membranes’ fatty acid profile. A higher concentration of AgNPs decreased the proportion of the unsaturated fatty acid (UFA) n-3 series, resulting in a decreased ratio of n-3UFA to n-6UFA.

Additionally, in this group, MUFA increased. The reduction of some MUFA and PUFA content (C20:1, C20:2, C20:3-n6) under the AgNP paint’s influence can also be explained by the oxidising effect of silver ions, which is indicated by an increase in TBARS content in the snail carcasses. Ebabe Elle [29] showed increased malonyl dialdehyde (MDA) levels in the livers as well as systemic inflammation, in rats fed with AgNPs in their diets at 500 mg/d/kg body weight. Intraperitoneal injection of 20 mg/kg silver lactate monohydrate induced lipid peroxidation in the liver and kidneys of mice [76]. As both the ionic forms and silver nano-particles show toxic properties, the ionic forms’ stronger negative effect has been confirmed [77]. 

Spraying the field with microorganisms, including bacteria and yeast, did not ultimately affect the transformation of fatty acids in the snails’ organism. The potential enrichment of the digestive system’s microbiota with bacteria and lactic acid yeast was not a sufficient catalyst for the conversion of fatty acids, as evidenced by the similar profile of these compounds in both this group of animals and in the control group. *Lactobacillus* strains have complex mechanisms by which different fatty acids are converted into shorter, longer, more saturated, or unsaturated fatty acids [78]. In poultry studies, an increased share of the sum of PUFAs, including linoleic and linolenic acid, was found in eggs after dietary supplementation with *Pediococcus acidilactici* [79]. In pig research, it was demonstrated that meat from a group of animals receiving *Lactobacillus amylovorus* and *Enterococcus faecium per os* was characterised by an increased content of MUFA and PUFA [78]. Although EM did not affect the fatty acid profile of the carcasses, in groups where two experimental factors were used, most of the changes in the fatty acid profile that were induced by the nano-Ag paint have either been negated by the addition of EM (palmitoleic acid, eicosenoic acid linoleic acid) or enhanced (palmitic acid, behenic acid, oleic acid, dihomo-γ-linolenic acid) in relation to the control group. In this group, it is noteworthy that the greatest amount of MUFA and eicosatrienoic acid was found due to the overlap of the effects of the two experimental factors. The latter deserves special attention since in the group exposed to the two research factors, the lowest share of α-Linolenic acid, a precursor of eicosatrienoic acid, was found [80]. 

## 5. Conclusions

The biosecurity strategies used in the experiment had a significant effect on the chemical composition of *Cornu aspersum aspersum* snails. A tendency for the fattening parameters and the retention of most elements to increase was observed in animals exposed to EM. However, EM did not affect the fatty acid profile of the carcasses. The paint containing the silver nano-particles became a factor that strongly remodelled the chemical composition of tissues of both minerals and fatty acids. Of particular note is the increased retention of zinc with the simultaneously increased retention of silver, which can be explained by the carrier proteins’ expression mechanism common to both elements. The increased degree of fatty acid oxidation in the same groups of animals and the remodelling of the fatty acid profile that resulted in reducing the 16- and 18-carbon acids and increasing the proportion of 20-carbon fatty acids, SFA, MUFA and PUFA, suggests that Ag influences the mechanisms of fatty acid retention or the elongation and oxidation mechanisms in which zinc participates. When assessing the benefits and negative effects of the applied biosecurity strategies, active microorganisms seem to have the best effect on the farm snails’ health and dietary values. In conclusion, snails can be a good model for the above-mentioned prevention methods in terms of benefits and risks to health and to the quality of products derived not only from invertebrates but other animals also. 

## Figures and Tables

**Table 1:** Proximate composition of snail carcasses (%) in the control and experimental groups.

Item	Control	Experimental Groups			SEM	*p*-Value
		AgNPs	EM	AgNPs + EM		
Dry matter [% FM]	16.13	19.93	24.03	22.16	1.729	0.098
Ether extract [% DM]	10.22 ^a^	10.30 ^a^	9.79 ^a^	12.05 ^b^	0.685	0.001
Crude protein [% DM]	52.29	58.51	54.19	54.68	4.384	0.984
Crude ash [% DM]	12.21	13.03	11.20	11.99	0.806	0.498

Control: feed tables covered by paint without silver nanoparticles; AgNPs: feed tables covered by paint containing the silver nanoparticles; EM: feed tables sprinkled by the water-based solution of Effective Microorganisms; AgNPs + EM: feed tables covered by paint containing the silver nanoparticles and sprinkled by the water-based solution of Effective Microorganisms. The means indicated with different superscripts (^a^, ^b^) are significantly different (*p* < 0.05). DM, dry matter; FM, fresh matter.

**Table 2:** The mineral content of snail carcasses (in dry matter) in the control and experimental groups.

Item	Control	Experimental Groups			SEM	*p*-Value
		AgNPs	EM	AgNPs + EM		
Ca [%]	2.08 ^b^	2.25 ^c^	2.17b ^c^	1.92 ^a^	0.043	0.000
Cu [mg/kg]	15.76 ^a^	16.39 ^ab^	18.64 ^b^	17.65 ^b^	0.415	0.015
Fe [mg/kg]	224.17 ^a^	261.81 ^b^	287.96 ^c^	253.57 ^b^	5.188	0.000
Mg [mg/kg]	3203.67 ^a^	3333.00 ^ab^	3623.34 ^c^	3417.51 ^b^	45.865	0.000
P [mg/kg]	12851.00 ^ab^	13587.90 ^bc^	13845.10 ^c^	12250.90 ^a^	257.441	0.001
Zn [mg/kg]	71.06 ^a^	113.72 ^d^	83.78 ^b^	91.30 ^c^	0.883	0.000
Ag [mg/kg]	0.06 ^b^	0.15 ^d^	0.05 ^a^	0.14 ^c^	0.002	0.0000

Control: feed tables covered by paint without silver nanoparticles; AgNPs: feed tables covered by paint containing the silver nanoparticles; EM: feed tables sprinkled by the water-based solution of Effective Microorganisms; AgNPs + EM: feed tables covered by paint containing the silver nanoparticles and sprinkled by the water-based solution of Effective Microorganisms. The means with different superscripts (^a^, ^b^, ^c^ and ^d^) are significantly different (*p* < 0.05).

**Table 3:** Fatty acid composition and TBARS content in the snail carcasses in the control and experimental groups.

Item		Control	Experimental Groups			SEM	*p*-Value
			AgNPs	EM	AgNPs + EM		
C14:0	Myristic acid	0.27	0.34	0.28	0.29	0.015	0.174
C16:0	Palmitic acid	7.51 ^a^	8.37 ^ab^	7.61 ^a^	8.79 ^b^	0.168	0.010
C17:0	Margaric acid	0.85	0.99	0.82	0.84	0.054	0.101
C18:0	Stearic acid	12.85	10.98	12.94	12.47	0.702	0.112
C20:0	Arachidic acid	0.75	0.77	0.75	0.84	0.055	0.234
C22:0	Behenic acid	1.42 ^b^	1.17 ^a^	1.42 ^b^	1.03 ^a^	0.040	0.000
Ʃ SFA		23.66	22.62	23.83	24.26	1.048	0.646
C16:1	Palmitoleic acid	0.36 ^a^	0.56 ^b^	0.32 ^a^	0.45 ^ab^	0.012	0.000
C18:1	Oleic acid	23.76 ^a^	26.61 ^a^	23.46 ^a^	28.66 ^b^	0.830	0.000
C18:1 t	Vaccenic acid	0.30	0.28	0.31	0.31	0.013	0.213
C20:1	Eicosenoic acid	3.58 ^b^	2.66 ^a^	3.58 ^b^	3.14 ^a^	0.118	0.005
Ʃ MUFA		28.02 ^a^	29.11 ^a^	27.68 ^a^	32.56 ^b^	0.655	0.002
C18:2-n6	Linoleic acid	25.34 ^a^	32.22 ^b^	24.98 ^a^	27.49 ^a^	0.868	0.006
C18:3-n3	α-Linolenic acid	1.86	1.33	1.13	0.95	0.556	0.223
C20:2	Eicosadienoic acid	9.48 ^b^	6.67 ^a^	9.66 ^b^	6.57 ^a^	0.404	0.000
C20:3-n3	Eicosatrienoic acid	0.46 ^a^	0.42 ^a^	0.49 ^a^	0.71 ^b^	0.026	0.000
C20:3-n6	Dihomo-γ-linolenic acid	5.97 ^b^	4.38 ^a^	6.21 ^b^	3.44 ^a^	0.289	0.000
Ʃ PUFA		43.34	45.23	42.69	39.36	2.028	0.088
TBARS (nmol/mg fresh matter)		0.11 ^a^	0.17 ^b^	0.11 ^a^	0.15 ^b^	0.006	0.000

Control: feed tables covered by paint without silver nanoparticles; AgNPs: feed tables covered by paint containing the silver nanoparticles; EM: feed tables sprinkled by the water-based solution of Effective Microorganisms; AgNPs + EM: feed tables covered by paint containing the silver nanoparticles and sprinkled by the water-based solution of Effective Microorganisms. SFA, saturated fatty acids; MUFA, monounsaturated fatty acids; PUFA, polyunsaturated fatty acids; Ʃ: sum. The means with different superscripts (^a^, ^b^) are significantly different (*p* < 0.05).

## Data Availability

Data available on request due to restrictions of privacy.
